# Single Versus Multiple Fractions of Palliative Radiotherapy for Bone Metastases: A Randomized Clinical Trial in Iranian Patients

**DOI:** 10.3747/co.v15i3.203

**Published:** 2008-06

**Authors:** F. Amouzegar–Hashemi, H. Behrouzi, A. Kazemian, B. Zarpak, P. Haddad

**Affiliations:** Radiation Oncology Department, Cancer Institute, Tehran University of Medical Sciences; Radiation Oncology Department, Bessat Hospital; and Statistics and Mathematics Department, Shahed University, Tehran, Iran

**Keywords:** Bone metastases, fractionation, palliative, radiotherapy

## Abstract

**Background:**

Despite high-level evidence in the literature, the use of single-fraction radiotherapy (rt) for management of painful bone metastases is not widely practiced in the world, as highlighted in several practice-pattern surveys. Fractionation of palliative rt for bone metastases has not been addressed in Iran, where the most common clinical practice is the use of 30 Gy in 10 fractions. Thus, we decided to perform a randomized clinical trial to compare responses in our patients with those reported in the international literature.

**Patients and Methods:**

Adult patients with multiple painful uncomplicated bone metastases were randomized to 8 Gy in a single fraction or 30 Gy in 10 fractions. Pain was graded by the patients on a scale of 1 to 4 just before and again 1 month after the end of rt. Palliative response was defined as “complete” (pain reduction of 2 grades or more), “partial” (pain reduction of 1 grade or more, but less than 2 grades), and “no response” (pain reduction of less than 1 grade).

**Results:**

We randomized 70 patients in this trial (63% women; mean age: 51.6 years). Sites of treatment included spine (*n* = 27), sacrum or pelvis (*n* = 25), extremities (*n* = 14), ribs (*n* = 3), and sternum (*n* = 1). Patients graded their pain before rt in a range from 1.8 to 4.0 (mean: 3.2). All patients finished their scheduled course of rt without incident.

Unfortunately, 5 patients died less than 1 month after the end of rt, and 7 did not return for any follow-up and could not be contacted. As a result, only 58 patients (31 who received multiple fractions, and 27 who received a single fraction) were available for evaluation of pain 1 month after treatment. At that time, pain was graded in a range from 1.0 to 4.0 (mean: 2.0). The reduction in pain grade ranged from –0.8 to 2.6 (mean: 1.1). We observed 8 (14%) complete responses, 33 (57%) partial responses, and 17 (29%) no responses, for an overall response rate of 71%.

The number of responders was 21 (78%) among those who received a single fraction and 20 (65%) among those who received multiple fractions (*p* > 0.1). The mean reduction in pain was 1.1 in both groups. The 10-fraction group contained a higher number of complete responders (11 of 31 as compared with 6 of 27 in the 1-fraction group)—a result that was not statistically significant. The mean reduction in pain was 1.4 in patients 50 years of age or younger and 0.9 in patients more than 50 years of age (*p* = 0.01). Of the 8 complete responses, 7 (87.5%) were seen in the patients 50 years of age or younger, and the mean age of patients with a complete response (38.7 years) was significantly lower than that of patients with a partial response or no response (53.7 years, *p* = 0.017).

By logistic regression, patient sex, primary tumour, rt site, and type of treatment (single-fraction vs. multifraction) did not have any significant effect on pain reduction. The only factor with a significant effect was age (*p* = 0.002).

**Conclusions:**

Our trial showed no significant difference in pain relief after palliative radiotherapy with 1 or 10 fractions in Iranian patients. The overall response rate was 71%, similar to results in the international literature. Younger patients responded better.

